# Nitrogen, or Azote

**Published:** 1859-01

**Authors:** N. R. Wright


### AMERICAN JOURNAL

OF

### DENTAL SCIENCE.

Vol. IX. NEW SERIES—JANUARY, 1859. No 1.

### ORIGINAL COMMUNICATIONS.

### ARTICLE I.

[Continued from the October Number.]

*Nitrogen, or Azote.* By Prof. N. R. Wright, M. D.

Symbol N. Eq. 14. S. G. 0.976.

This gas was first noticed, though in a cursory manner, by the celebrated Dr. Rutherford, of Edinburgh, in the year 1772, who during some experiments upon the results of animal respiration, found that after removing carbonic acid with lime water, a substance remained which would not support combustion. Lavoisier and Scheele in the year 1775, independently of each other, discovered it to be a constituent of the atmosphere in the proportion of four-fifths. Lavoisier, on account of its unfitness for respiration, called it azote from α primitive, and ζωη life, and when it was ascertained to be a constituent of nitric acid, Chaptal called it nitrogen.

Nitrogen exists in nearly all animal substances, but is found in very few vegetables.

*Preparation.* It may be obtained by passing chlorine gas through ammonia, which is decomposed, yielding its hydrogen to unite with chlorine, forming hydro-chloric acid, while the liberated nitrogen comes over.

2. A ready method of obtaining it pure, is to pass a current of atmospheric air over small fragments of copper, contained in a tube and heated to redness, the oxygen is all absorbed, leaving nitrogen.

3. A piece of phosphorus placed under a bell glass of air, and allowed to remain for a few days, will remove all the oxygen, leaving nitrogen, contaminated however with a little phosphorus vapor and carbonic acid, which may be got rid of by washing with a little lime water, or a solution of potash.

4. The method most generally adopted is to float a porcelain saucer, containing a piece of phosphorus, upon the water of a pneumatic trough; having ignited the phosphorus, cover with a clean bell glass containing atmospheric air; the combustion will continue until all the oxygen has been removed, and the glass will be found to contain nitrogen mixed with the white fumes of phosphoric acid, and a little *carbonic acid;* the former, from its strong affinity for water, is rapidly absorbed by that liquid—a half hour sufficing to remove it completely from a large jar, even when there is no agitation; and passing the gas rapidly from one jar to another, will accomplish the same result in a few minutes; carbonic acid may be removed by washing with potash or lime water, as mentioned above.

*Properties.*—By whatever method obtained, pure nitrogen is colorless, tasteless, inodorous and lighter than atmospheric air, having a specific gravity of 0.976. It seems to have very few affinities, uniting directly with but few substances even under the most favorable circumstances; among these are oxygen, carbon and hydrogen.

Nitrogen is a gas of negative rather than positive qualities, and seems to be mixed with oxygen in the atmosphere, more for the purpose of retarding oxydation and combustion, than with any other view; again, an atmosphere of pure oxygen would be entirely too stimulating for prolonged respiration, and nitrogen with its negative properties dilutes it until it becomes a safely respirable medium. Animals placed in an atmosphere of nitrogen die, not because the gas itself is poisonous, like chlorine and others, but because of the absence of oxygen, which is indispensable for the decarbonization of the blood, and without which respiration would be imperfectly carried on for a very limited period. A lighted taper immersed in this gas, is instantly extinguished, without leaving a spark on the wick.

When mixed with oxygen in a suitable vessel, containing a little water, the two gases may be made to unite so as to form nitric acid, by passing through them a number of electric sparks.

* "The water formed by the combustion of hydrogen in air, or of a mixture of hydrogen and nitrogen in oxygen, has often an acid reaction, which is due to a trace of nitric acid. But when the hydrogen is mixed with air in excess, so as to prevent great elevation of temperature during the combustion, the oxydation of the nitrogen does not take place."

When alkalies are present, nitric acid is frequently formed in considerable quantities, combining with the alkalies forming salts, which are found mixed with the soils in many localities, it is in this way that "nitre soils" on artificial nitre beds, (as they are called,) are produced.

### *Compounds with Oxygen.*

Nitrogen forms with oxygen five *chemical* compounds, and a sixth, which is now almost universally regarded as a mechanical mixture. The following is a list of them with their chemical constitution expressed by symbols:

* Graham, page 323, Eng. Ed.

| | | |
|---|---|---|
| | Atmospheric air, . . | N 80 or 79 to O 20 or 21 |
| 1. | Protoxyd of nitrogen, | $= N + O = 22$ — |
| 2. | Binoxyd of nitrogen, | $= N + O_2 = 30$ — |
| 3. | Nitrous acid, . . . | $= N + O_3 = 30$ — |
| 4. | Peroxyd of nitrogen, or hyponitric acid, | $= N + O_4 = 46$ — |
| 5. | Nitric acid, . . . | $= N + O_5 = 54$ — |

Atmospheric air is the mixture which surrounds our globe, extending to an indefinite distance above its surface, its temperature and density constantly decreasing with every increase of elevation. When the thermometer indicates a temperature of 60° F., the atmosphere at the level of the sea is capable of sustaining a column of mercury at the height of 29-8 inches, or a column of water thirty-four feet, pressing with a force of fifteen pounds to the square inch.

That the temperature of the atmosphere varies with the elevation, has been repeatedly proven by actual observation, on the tops of high mountains and at great elevations in balloons, a fact which is easily explained when we take into account the circumstances that the atmosphere does not receive its heat from direct solar radiation, and that rarified air or gases have a greatly diminished capacity for heat.

The sun's rays pass through the atmosphere, and falling upon the surface of the earth, impart their heat to that body; those portions of the superincumbent air, in contact with it, receive a part of the heat, and thus becoming specifically lighter, ascend, giving place to cool and heavier portions, which, becoming heated in their turn, rise also. Thus it will be perceived that the atmosphere cannot be heated in the course of an ordinary day, to a very great height above the earth's surface; and it must be obvious also, that the temperature must diminish rapidly from the earth upwards.

Again, the atmosphere is most dense at the surface of the earth, diminishing rapidly in density as we ascend above it; as compression increases the capacity of air for heat, and rarefaction diminishes it, it follows that the greater the elevation above the earth's surface, the less will be the capacity of air for heat; this is found to be actually true, the rate of diminution being about 1° F. for every 350 feet.

If the above statement can be relied on, there must be a point in every latitude, where the temperature never gets above 32°—a point of perpetual congelation—highest at the equator, and gradually diminishing to the north and south poles, where it is probably at the surface of the ground.

Atmospheric air owes to the presence of oxygen its principal chemical qualities, being nearly or quite negative in all its properties, when that gas is removed from it; it will not support animal life, nor will combustibles burn in it, metals and other oxydable bodies are not affected even when strongly heated in contact with it, and changes which occur in most organic substances in the presence of atmospheric air under ordinary circumstances, do not here take place. Besides acting as a diluent of oxygen in the atmosphere, nitrogen plays an important part in the animal system, which, however, can scarcely be said to have been satisfactorily demonstrated. Many careful attempts have been made, from time to time, for the purpose of deciding whether or not the atmosphere really is a *chemical compound*, or merely a *mechanical mixture*, and although we cannot make any positive assertion either way, the probabilities are that it belongs to the latter class.

Several circumstances would induce us to believe that air is a veritable and regular chemical compound, among which we find the following worthy of notice. The two gases have different specific gravities, and in obedience to the law of equilibrium of fluids having different densities, we might expect in a mere mixture, the heavier oxygen to subside to the bottom of the atmosphere, leaving the nitrogen floating in the upper regions above it; this, however, has never been observed under any circumstances.

Many attempts have been made to occasion the separation by difference in gravity of the two gases, in tubes constructed for the purpose, but they have all failed; again, they have been placed separately in tubes in the order of their densities, and after standing a few hours, have been found thoroughly mixed in all parts of them.

The strongest argument in favor of the truly chemical constitution of air, is the fact, that the component gases are united in definite proportions both by volume and weight, thus:

By volume—1 oxygen + 4 nitrogen.

By weight—1 oxygen + 2 nitrogen.

Though all these, and more very plausible arguments can be adduced in favor of the chemical hypothesis, as many equally strong can be adduced against it, sufficient, if not to establish the mechanical theory, at least to throw considerable doubt on the other view.

The facility with which oxygen can be separated from the combination, is almost unexampled in the history of chemical combinations, any substance having an affinity for it, being sufficient to effect its removal. A stick of phosphorus, immersed for a short time in a vessel containing air, will appropriate all the oxygen, every instance of combustion and respiration produces a like result, even rain drops will absorb it in their passage through the air to the amount of 20 or 25 per cent.

Its power of refracting light is what we ought to expect from a volumetrical mixture, and not a chemical compound.

When the two gases are mixed in the proportions of 1 oxygen to 4 nitrogen, by volume, we have a compound possessing all the qualities of pure atmospheric air; and unlike most chemical combinations, without change of bulk or other qualities possessed by the mixture before their union.

As I do not propose to enter into a detail of the physical properties of the air, I will conclude the subject with some remarks on the mode by which its composition is kept uniform. Oxygen is so important for many purposes in the economy of nature, as for example, *respiration*, *combustion*, &c., and such immense quantities are hourly consumed that were there not some compensating process, the atmosphere would speedily become unfit for its uses; now let us see how this compensation is accomplished.

According to Turner, page 273, Eng. Ed., "the only source by which oxygen is known to be supplied, is the action of growing vegetables. A healthy plant absorbs carbonic acid during the day, appropriates the carbonaceous parts of that gas to its own wants, and evolves the oxygen with which it was combined. During the night indeed, an opposite effect is produced. Oxygen gas then disappears, and carbonic acid is eliminated; but it follows from the experiments of Davy, Priestly and Daubeny, that plants during twenty-four hours yield more oxygen than they consume. Whether living vegetables make a full compensation for the oxygen removed from the air, by the processes above mentioned, is uncertain. From the great extent of the atmosphere, and the continual agitation to which its different parts are subject by the action of winds, the effects of any deteriorating process would be very gradual, and a change in the proportion of its elements could be perceived only by observations made at very distant intervals."

### PROTOXYD OF NITROGEN.

Symbol—N O. Eq. 22

*History.*—This gas was discovered by Priestly in 1776, and called by him dephlogisticated air, but for the most minute and careful examination of its properties we are indebted to Sir H. Davy, who published in the early part of the present century his "Researches on the Nitrous Oxyd;" to him also we are indebted for the first mention of its exhilerating properties.

*Preparation.*—There are several modes by which this gas may be obtained, but as the *nitrate* of *ammonia* process is the simplest and most generally used, I propose to give only that. The salt in question may be purchased already prepared, but I would recommend its preparation in the laboratory, which may be done in the following manner: Pour into a large evaporating basin from eight to sixteen ounces of nitric acid, and drop into this lumps of carbonate of ammonia as long as effervescence continues, indeed, it is better to add ammonia in slight excess ; evaporate the solution until a drop let fall upon a cool surface will solidify, whereupon the basin may be set aside until its contents are cold—it will be found now to contain a solid mass of nitrate of ammonia, which is to be broken up into small fragments and bottled away for use; the salt being very deliquescent, the atmosphere should be excluded as much as possible.

If put into a flask or retort, and exposed to the action of heat, it will fuse below 400°, and when the temperature reaches about 500°, decomposition takes place ; and if heat be continued sufficiently long, all the salt will disappear, having been resolved (if pure) into *nothing* except *water*, and *protoxyd* of nitrogen—the latter being received over water heated to 90°, because of the waste occasioned through absorption by cold water. Should the flask become partially filled with white fumes, diminish the heat immediately, in fact, from the beginning of the process, ebulition should not be allowed to become violent, for if it is, little besides nitrogen comes over.

The rationale of the process is as follows, the nitric acid and ammonia are both decomposed, and we have, as the result of the decomposition, four atoms of water and two of protroxyd of nitrogen, or exhilerating gas, the formula by symbols is thus expressed:

$$NH_3, HO + NO_5 = 2\,NO + 4\,HO—$$

It may, perhaps, be more distinctly understood by inspection of the following formula, which will be found in the English edition of Graham's Chemistry, page 339.

| Before Decomposition. | | After Decomposition. | |
|---|---|---|---|
| 80 Nitrate of Ammonia, | 54 N. Acid, | Oxygen, | 22 N. Oxyd. |
| | | Oxygen, | 22 N. Oxyd. |
| | | Oxygen, | |
| | | Oxygen, | |
| | | Oxygen, | |
| | | Nitrogen, | |
| | 17 Ammonia, | Nitrogen, | |
| | | Hydrogen, | Water. |
| | 9 Water, | Hydrogen, | Water. |
| | | Hydrogen, | Water. |
| | | Water, | Water. |
| 80 | 80 | 80 | 80 |

*Properties.*—This gas has many of the properties of pure oxygen, but all of them are exhibited in a less degree. It supports combustion quite freely, all substances which burn in ordinary atmospheric air, burning in it with greater splendor, nitrogen being left after the combustion is accomplished. Mixed with hydrogen in equal bulks, it explodes by the electric spark, or by contact with a lighted taper.

The most remarkable property possessed by this gas is the effect produced upon the animal system when it is respired: it may be taken into the lungs with perfect impunity, producing an agreeable intoxication, which lasts for a few minutes, and then passes off, leaving no unpleasant sensations whatever, in the majority of instances.

The best method of administering the gas, is as follows: An India rubber bag or large bladder is emptied of air, and filled with the gas, which (after all the air contained in the lungs has been expelled) is respired for the space of half a minute to a minute, by which time, usually, the effect is produced; varying with different individuals, and at different times with the same individual, and but seldom producing any unpleasant effects.

### BINOXYD OF NITROGEN, OR NITRIC OXYD.

Symbol $NO_2$ Eq. 30.

*History.*—This substance was a discovery of Dr. Hales, but to the investigations of Dr. Priestly we are indebted for the more minute examination of its properties.

*Preparation*—It is easily obtained by the action of diluted nitric acid upon many of the metals, but of all, *copper* and *mercury* are the best, yielding a purer gas; mercury, however, requires a little heat, and in this respect, copper, which requires none, has the advantage. Cut up some strips of sheet copper, throw them into an ordinary gas bottle or retort, and pour upon them some weak nitric acid; action immediately commences, and brisk effervescence continues until all the materials are consumed—the gas, meantime, having been collected at an ordinary pneumatic trough.

A part of the acid in this process is decomposed, and oxyd of copper is formed, and the remaining undecomposed nitric acid combines with the oxyd, forming nitrate of copper, which having a deep blue color, imparts the same to the liquid. Upon subsequently evaporating this blue solution we obtain a large crop of beautiful blue crystals, of a highly deliquescent salt, which upon examination proves to be nitrate of copper.

Perhaps the decomposition which occurs in this instance may be more easily comprehended by a formula showing the component parts separately.

Nitric acid consists of

| | |
|---|---|
| | Nitrogen, |
| Nitric Acid, | Oxygen, |
| | Oxygen, |
| | Oxygen, |
| | Oxygen, |
| | Oxygen. |

A portion of this acid is decomposed into its constituent parts—two equivalents of oxygen remain combined with the nitrogen, forming *binoxyd of nitrogen,* while the other three equivalents of oxygen combine with the copper, forming three equivalents of oxyd of copper, which, at the same time, combining with the nitric acid forms nitrate of copper ; thus—

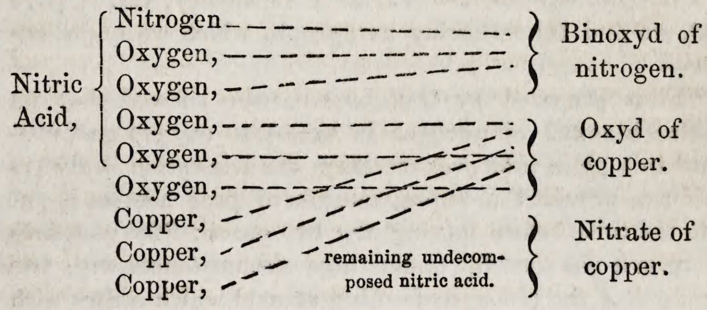

*Properties.*—When obtained where atmospheric air cannot gain access, this gas is transparent and colorless, but has an intensely disagreeable odor, and if only a very small portion enters the lungs, is extremely irritating; mixed with atmospheric air, dense orange colored fumes immediately make their appearance, resulting from the formation of *peroxyd of nitrogen,* and the affinity thus evinced for oxygen is perhaps the most singular property of the gas ; indeed, so strong is its attraction that it will abstract oxygen completely from almost any of its combinations. It has been employed on this account in atmospheric analysis, but owing to the amount of contraction which occurs, the absolute amount of oxygen which has disappeared, cannot be accurately ascertained. Its effect upon combustion is somewhat peculiar, some combustibles, as phosphorus, charcoal, &c., introduced in a state of ignition, burning with increased splendor, while a lighted taper, &c., will be extinguished.

Binoxyd of nitrogen, or nitric oxyd, consists of one equivalent of nitrogen and two of oxygen, and is expressed symbolically by $N + O_2$, or $NO_2$.

### NITROUS ACID—FORMERLY HYPONITROUS ACID.

Symbol $NO_3$, or $N + O_3 = $ Eq. 38.

*History.*—This substance was first discovered and described by *Gay Lussac*, and subsequently carefully examined by *Thenard*, who gave it the name of *azotous acid.*

*Preparation.*—In Dr. Turner's Chemistry, vol. 1, page 280, we find the following paragraph, which we quote entire—

"First prepared by Gay Lussac, who showed that on adding binoxyd of nitrogen in excess to oxygen gas, confined in a glass tube over mercury, the absorption is always uniform, provided a strong solution of pure potassa is put into the tube before mixing the two gases; fifty measures of oxygen gas combine under these circumstances with two hundred of the binoxyd, forming an acid which unites with the potassa. As the binoxyd contains half its volume of oxygen gas, the new acid must be composed of one hundred measures of nitrogen, and one hundred and fifty of oxygen.

"It is generated when the binoxyd is kept for a considerable time say three months, in a glass tube over mercury, with a strong solution of pure potassa, when the binoxyd is resolved into hyponitrous acid, (nitrous acid) which unites with the *alkali*, while protoxyd of nitrogen remains in the tube; and Dulong formed it by mixing two hundred measures of binoxyd of nitrogen with fifty of oxygen gas, both quite dry, and exposing the resulting orange fumes to intense cold, which condensed them into a liquid. *It is the* NITROUS ACID *of Berzelius and other continental chemists.*"

*Properties.*—Nitrous acid, when perfectly formed, has, under ordinary circumstances, and at ordinary temperatures, a bright green color, volatilizing very readily into a yellowish colored vapor; decomposing at once when thrown into water, nitric acid being formed, and binoxyd of nitrogen escaping with effervescence.

This substance combines with several acids, as nitric, sulphuric, &c., but cannot be made to combine directly with alkalies; earths, &c. except under peculiar circumstances. If we mix a considerable excess of binoxyd of nitrogen with oxygen, over a strong solution of pure potash, the gases will be found to disappear in the proportions of the nitrous acid produced, and the latter combines with the potash, forming *nitrate of potash.*

"Nitrites of the alkalies, and alkaline earths may be obtained by heating the corresponding *nitrates* to a gentle red heat; and the *nitrite* of the *oxyd of lead* is formed by boiling a solution of the *nitrate* of that *oxyd* with *metallic lead.*"*

Mitscherlich has proposed the formation of the *nitrite of soda,* and from this the production of *nitrite of silver* by precipitation; this is to be purified by solution and crystalization; with this salt it is proposed by the aid of double decomposition, to produce the other nitrites. Alcohol dissolves the nitrites of potash and soda.

### PEROXYD OF NITROGEN.

Symbol, $NO_4$, or $N + O_4 = \text{Eq. } 46$.

*History.*—This substance is formed whenever binoxyd of nitrogen and oxygen, or atmospheric air are mixed, and its production is always manifest by the appearance of dense orange colored fumes in the vessel in which the mixture occurs. We prefer calling it *peroxyd* of nitrogen, rather than *hypo*-nitric acid, because it has none of the prominent characteristics of acids, such as union with bases for the formation of salts, &c.

*Preparation.*—Though it may be prepared by mixing its constituent gases in certain proportions, the most reliable method is the distillation of *nitrate of lead,* in a retort of refractory glass, at a red heat, the substance formed being liquified in a receiver, kept cold by being surrounded with a mixture of ice, or snow and salt. The nitric acid in this process, is decomposed—one equivalent of its oxygen being

* **Turner, page 280, vol. 1.**

driven off while four remain in combination with the nitrogen.

Nitric acid

$\overbrace{N + O_5}$ being decomposed yields $NO_4 + O$.

*Properties.*—Under ordinary circumstances, *peroxyd of nitrogen* is a reddish colored liquid, but undergoes great changes in appearance from alterations in temperature. Below zero, say 40°, it becomes transformed into a white solid, it is a colorless liquid at or below zero, and passes through every gradation of yellow as the temperature rises, until at length when it becomes highly heated, it assumes a dark orange red color. A similar change in color is observed when water is added to it, but in the reverse order, as the amount of water added is increased, the liquid becomes more and more yellow, then changes to green and blue, and finally, it becomes entirely colorless. There is no known compound of peroxyd of nitrogen, though many curious speculations have been made as to its radical nature. Many of the metals decompose it with facility.

